# Association Mapping of Seedling Resistance to *Fusarium graminearum* Root Rot and Development of KASP Assays in Soybean

**DOI:** 10.3390/plants15162479

**Published:** 2026-08-16

**Authors:** Xiangkun Meng, Zhongqiu Fu, Wantong Zhao, Xu Wu, Chang Ma, Yanzeng Feng, Shibo Du, Xue Zhao, Yuhe Wang, Yingpeng Han

**Affiliations:** Key Laboratory of Soybean Biology in Chinese Education Ministry, Northeast Agricultural University, Harbin 150030, China; 15647065179@163.com (X.M.); fzq7743@163.com (Z.F.); zwtong1688@163.com (W.Z.); wuxubio@163.com (X.W.); changma_deyouxiang@163.com (C.M.); fyz1763744208@163.com (Y.F.); dsb9832026@163.com (S.D.); xuezhao@neau.edu.cn (X.Z.)

**Keywords:** soybean, *Fusarium graminearum*, genome-wide association study, KASP

## Abstract

Soybean root rot caused by *Fusarium graminearum* is an important soil-borne disease. It hinders seedling establishment and ultimately reduces soybean yield. Resistant germplasm and reliable molecular markers are therefore needed for resistance breeding. In this study, 336 soybean accessions were evaluated for resistance to *F. graminearum* root rot using the disease severity index (DSI), which ranged from 5.71 to 100.00 across the association panel. Genome-wide association analysis was performed using resequencing-based single nucleotide polymorphism (SNP) data with mixed linear model (MLM) and Fixed and random model Circulating Probability Unification (FarmCPU) models, which detected 117 and 113 candidate resistance-associated SNPs, respectively. Among these, 105 shared SNPs were used to define candidate genomic intervals containing 247 annotated genes. Based on Gene Ontology (GO) and Kyoto Encyclopedia of Genes and Genomes (KEGG) enrichment, functional annotation, and allelic-effect analysis, six candidate genes and their associated exonic SNPs were prioritized. Quantitative reverse transcription polymerase chain reaction (qRT-PCR) analysis showed infection-responsive expression patterns for all six candidate genes, with *Glyma.17g202500* and *Glyma.18g266700* showing stronger induction in the resistant accession. Two SNPs in these genes were converted into Kompetitive allele-specific PCR (KASP) assays. KASP-S17_32244510 and KASP-S18_55105706 were successfully developed for genotype screening, with screening efficiencies of 73.08% and 74.29%, respectively. These findings identify useful genetic targets and molecular markers for improving soybean resistance to root rot caused by *F. graminearum*.

## 1. Introduction

Soybean (*Glycine max* (L.) Merr.) is cultivated worldwide as a major source of plant protein and edible oil [1]. According to the latest available agricultural production statistics reported by the Food and Agriculture Organization of the United Nations (FAO), global soybean production reached approximately 398 million tonnes in 2024 [2]. Soybean root rot may occur throughout plant development. Affected plants often exhibit root discoloration and tissue decay, followed by poor stand establishment, wilting, and reduced yield [3,4]. *Fusarium* spp. are common pathogens of soybean root rot and occur widely in soybean-producing regions [5]. Among these fungi, *F. graminearum* is an important pathogen associated with soybean root rot. It can infect seeds and young plants, leading to seed decay, damping-off, and root rot [6]. The disease is of particular concern in temperate production regions, where maize–soybean rotation can promote pathogen survival and cross-crop infection [7]. In China, soybean production is concentrated largely in the northeastern region, where *Fusarium* root rot is an important soil-borne disease. In Heilongjiang Province, soybean root rot has been reported with disease incidence ranging from 75% to 90% and yield losses of approximately 10–20% [8,9]. Under favorable environmental conditions, disease severity can exceed 90% in greenhouse experiments [10]. In the United States, soybean seedling diseases have caused average annual losses of $21.8 million over the past five years, with *F. graminearum* representing an important component of this disease complex [11]. Although fungicidal seed treatments, soil management, and crop rotation are commonly used, their protection is often limited, and repeated fungicide use may select for resistant pathogen populations [12,13]. Therefore, identifying resistant germplasm and developing reliable molecular markers are important for improving soybean resistance to *F. graminearum* root rot (FGRR).

In soybean, genome-wide association studies (GWAS) are commonly used to link natural genetic variation with complex trait performance across diverse germplasm panels [14]. They have also helped uncover genomic regions involved in disease resistance, including loci underlying resistance to sudden death syndrome [15]. In *Fusarium* root rot, association mapping has provided useful genetic information. For example, a GWAS involving 350 soybean accessions revealed eight single nucleotide polymorphisms (SNPs) linked to resistance to *F. oxysporum,* with the strongest association signals located on chromosome 6 [16]. For *F. graminearum*, existing studies suggest a quantitative and genetically complex pattern of resistance. The quantitative and complex nature of *F. graminearum* resistance is supported by an SLAF-seq-based GWAS of 314 soybean accessions, which detected 12 resistance-associated SNPs across eight chromosomes, including one overlapping qRfg_Gm06 and several novel loci [10]. Linkage mapping studies have further identified Quantitative Trait Loci (QTLs) on chromosomes 6, 8, 13, 15, 16, and 19 in different recombinant inbred line populations [17,18,19]. Among these loci, qRfg_Gm06 was fine-mapped to an approximately 400 kb interval on chromosome 6, and a KASP assay was developed for one candidate gene within this region [7]. More recently, a novel QTL explaining 20.29% of the phenotypic variation in post-emergence disease severity was detected in an F_2:3_ population derived from PI 438500 × PI 548631 [11]. Comparative GWAS across multiple *Fusarium* species also found no common candidate genes across all pathogens, suggesting that resistance to different *Fusarium* species may involve pathogen-specific genetic mechanisms [20,21]. Previous studies collectively indicate that soybean resistance to *F. graminearum* has a polygenic basis. The loci detected for this trait can differ among germplasm panels, pathogen isolates, and evaluation systems. This variation supports the need for further association mapping in diverse soybean materials. It also highlights the value of converting reliable resistance-associated loci into markers that can be used for practical screening.

Molecular markers are widely used in crop genetic studies and germplasm evaluation. They are particularly useful for identifying trait-associated alleles and supporting marker-assisted selection [21]. Several types of markers have been used in crop improvement, but SNP-based assays are now more widely adopted because of their stability, scalability, and ease of genotyping [22]. Among them, Kompetitive allele-specific PCR (KASP) markers are particularly suitable for large-scale screening, as they are cost-effective, locus-specific, and compatible with high-throughput genotyping platforms [23]. KASP assays have been widely used to develop trait-linked markers in crop species. Applications include yield and stress tolerance in wheat, as well as adaptability and insect resistance in rice [24,25,26]. KASP-based genotyping has also been reported in soybean for seed composition traits, low Kunitz trypsin inhibitor content, nematode resistance, and resistance to *F. oxysporum* root rot [27,28,29]. For FGRR resistance, KASP markers developed from the qRfg_Gm06 locus have been applied in recombinant inbred populations [7]. Nevertheless, available markers for FGRR resistance remain limited, especially those tested in diverse soybean germplasm [30]. Developing additional resistance-associated SNP markers is therefore still needed to support efficient screening and breeding of resistant soybean materials.

To address this need, we first phenotyped 336 soybean accessions for their seedling response to *F. graminearum* root rot. Disease grades were assigned according to symptom severity and then used for resistance classification and disease severity index (DSI) calculation. Resequencing-derived SNPs were then used for GWAS with both mixed linear model (MLM) and Fixed and random model Circulating Probability Unification (FarmCPU) models, and the shared association signals were used to define candidate genomic regions. Genes within these regions were further examined by functional annotation, allelic-effect comparison, and quantitative reverse transcription PCR (qRT-PCR) analysis in resistant and susceptible genotypes. Based on the association results, allelic effects, and expression patterns of the corresponding candidate genes, two SNPs were selected and converted into KASP markers. This study examined the genetic basis of FGRR resistance by identifying relevant genomic regions and candidate genes. It also aimed to develop practical markers for soybean germplasm evaluation and resistance breeding.

## 2. Results

### 2.1. Phenotypic Variation in Soybean Resistance to Fusarium graminearum Root Rot

**Figure 1:**
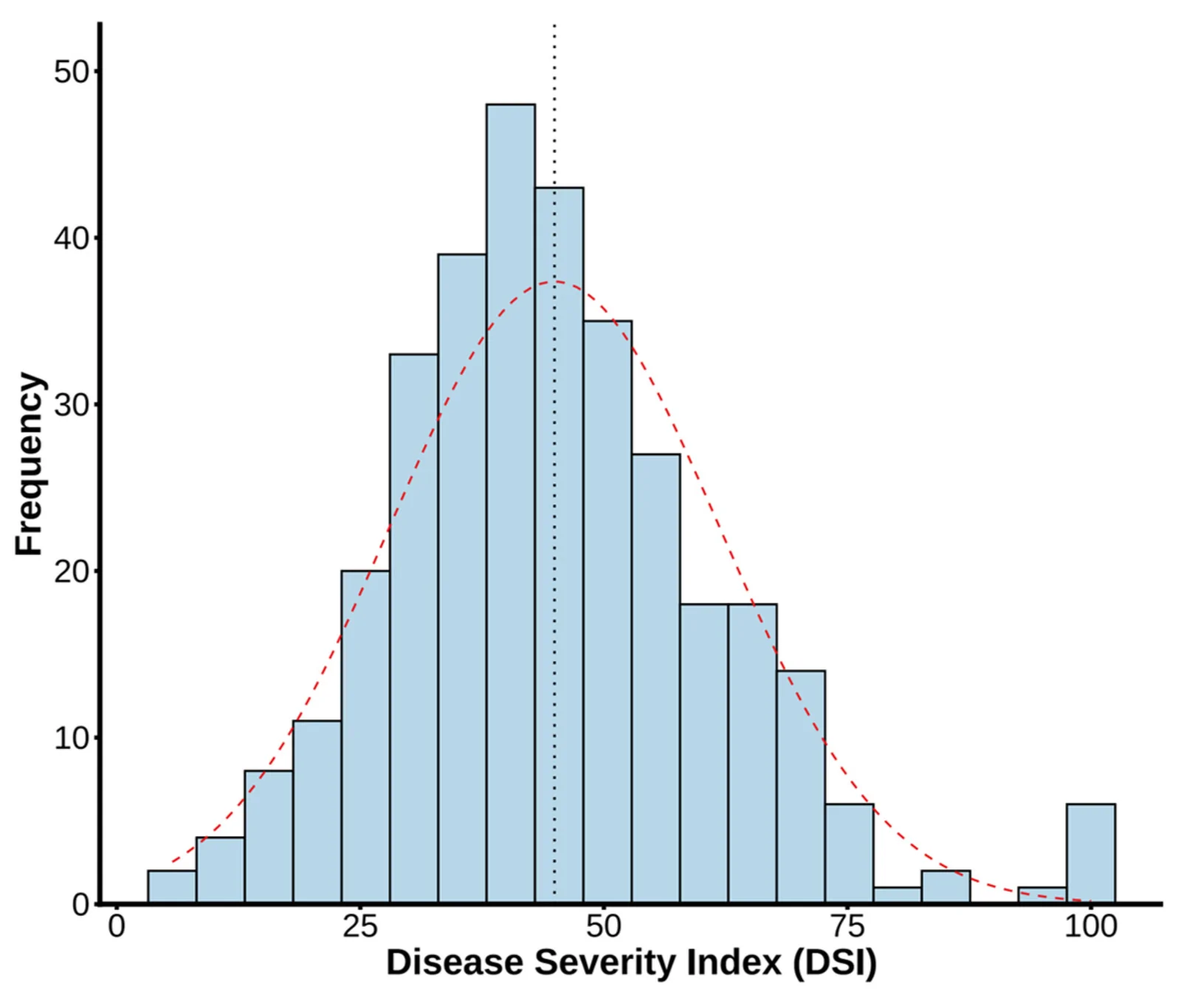
Frequency distribution of disease severity index (DSI) values among 336 soybean accessions following inoculation with *Fusarium graminearum*. The red dashed curve represents the fitted normal distribution, and the black vertical dotted line indicates the mean DSI value.

Disease response differed markedly among the 336 accessions, with DSI values covering a broad range from 5.71 to 100.00. Across the panel, the mean DSI was 44.93 ± 16.91. The distribution was continuous and approximately unimodal (Figure 1, Supplementary Table S1), indicating broad phenotypic variation for FGRR resistance within the association panel. According to the DSI-based classification criteria, no accession was classified as immune. The accessions were therefore distributed across four observed resistance categories. Seventeen accessions were highly resistant (HR, 0 < DSI ≤ 20), accounting for 5.06% of the panel. In addition, 119 accessions were resistant (R, 20 < DSI ≤ 40), 191 were susceptible (S, 40 < DSI ≤ 80), and 9 were highly susceptible (HS, DSI > 80), representing 35.42%, 56.85%, and 2.68% of the panel, respectively. These results indicate that the panel contained sufficient phenotypic variation for subsequent genome-wide association analysis.

### 2.2. Genome-Wide Association Analysis of FGRR Resistance in Soybean

The 336 soybean germplasm accessions were analyzed using SNPs generated from resequencing. SNPs were filtered for allele frequency and genotype completeness before GWAS, leaving 1,329,617 high-quality variants for subsequent analyses. Their distribution and density across the 20 soybean chromosomes are summarized in Supplementary Table S2. These variants covered all 20 soybean chromosomes. Examination of the first ten principal components revealed a clear inflection at PC3 (Supplementary Figure S1a), suggesting that the first three principal components captured the main population-structure pattern in this panel (Supplementary Figure S1b). Kinship analysis was also performed using all SNP markers. The pairwise kinship coefficients among the 336 soybean accessions were generally low, indicating weak genetic relatedness within the population (Supplementary Figure S1c).

**Figure 2:**
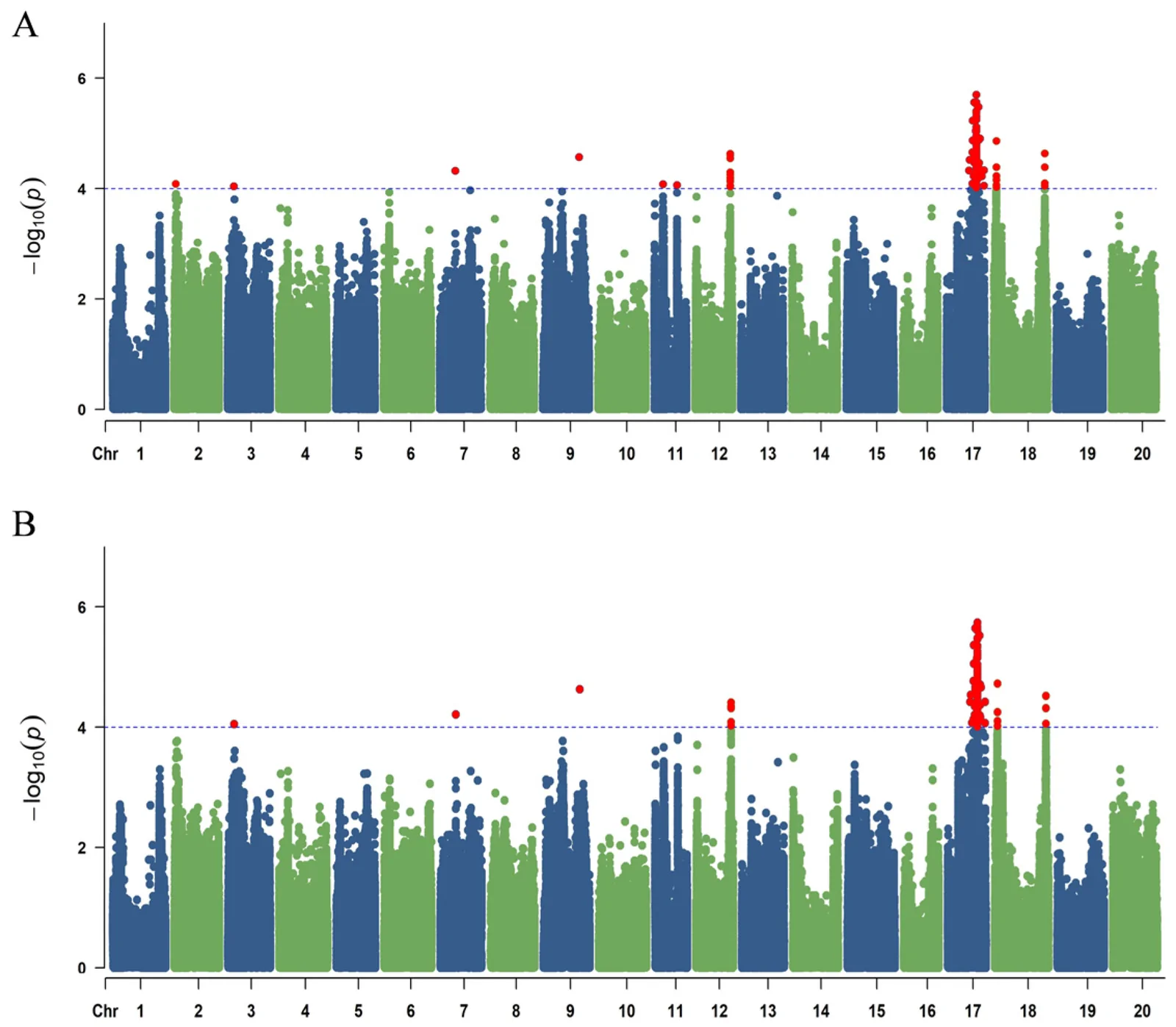
Manhattan plots of GWAS for FGRR resistance in soybean. (**A**) Mixed linear model (MLM). (**B**) FarmCPU model. Alternating blue and green colors represent different chromosomes, red points indicate SNPs exceeding the suggestive threshold, and the dashed line indicates this threshold.

Genome-wide association mapping was conducted to uncover loci underlying differences in DSI after *F. graminearum* infection. Variants reaching the suggestive significance level of −log_10_(*P*) ≥ 4.0 were carried forward for subsequent analyses. Using this threshold, the MLM detected 117 candidate resistance-associated SNPs distributed across 8 chromosomes (Supplementary Table S3). The strongest signal cluster was located on chromosome 17, with additional peaks detected on several other chromosomes, including chromosomes 2, 3, 7, 9, 11, 12, and 18 (Figure 2A). The FarmCPU model identified 113 candidate SNPs across 6 chromosomes (Supplementary Table S4). Similar to the MLM result, FarmCPU detected a major signal cluster on chromosome 17. Additional signals were observed on chromosomes 3, 7, 9, 12 and 18 (Figure 2B).

A comparison of the two models identified 105 SNPs that were detected by both MLM and FarmCPU. These shared SNPs accounted for 89.7% of the MLM-detected SNPs and 92.9% of the FarmCPU-detected SNPs, indicating a high degree of overlap between the two GWAS models. The overlapping SNPs were therefore retained as candidate association signals for subsequent interval definition and candidate gene analysis. The SNPs identified by each model are reported in Supplementary Tables S3 and S4, respectively.

### 2.3. Functional Annotation of Candidate Intervals and Identification of Candidate Genes

**Figure 3:**
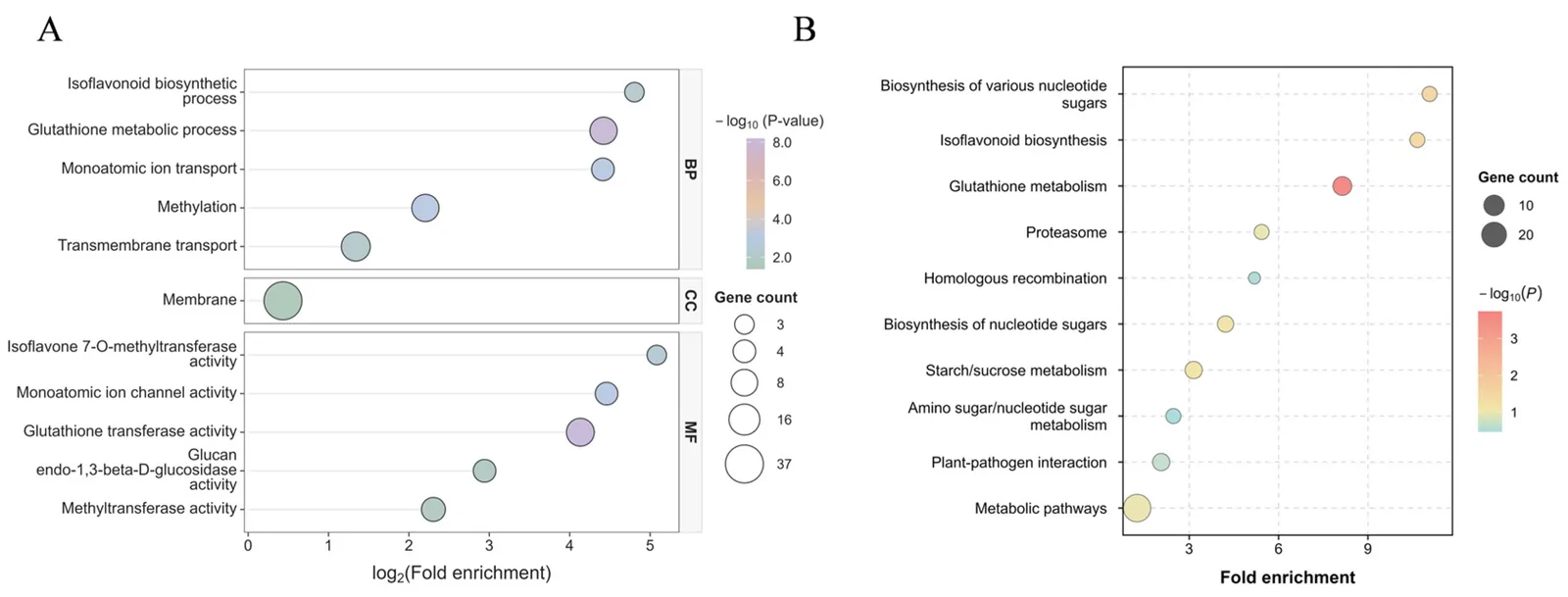
Functional enrichment analysis of genes within the candidate regions. (**A**) Enriched GO terms. (**B**) Enriched KEGG pathways.

Candidate genomic intervals were defined as 100 kb upstream and downstream of the 105 SNPs detected by both the MLM and FarmCPU models. Consolidation of overlapping intervals yielded 247 annotated genes in the Wm82.a2.v1 soybean reference assembly (Supplementary Table S5). Gene Ontology (GO) and Kyoto Encyclopedia of Genes and Genomes (KEGG) enrichment analyses were performed to explore the potential functions of genes within the candidate regions. GO terms were mainly related to isoflavonoid biosynthesis, glutathione metabolism, membrane-associated transport, and methylation. The enriched molecular functions were largely associated with transferase and ion channel activities, including isoflavone 7-O-methyltransferase activity, glutathione transferase activity, and monoatomic ion channel activity (Figure 3A). KEGG enrichment further indicated the involvement of these genes in isoflavonoid and glutathione metabolism, nucleotide sugar and carbohydrate metabolism, proteasome activity, homologous recombination, and plant–pathogen interaction (Figure 3B). Overall, the enrichment patterns suggest that the candidate genes may contribute to secondary metabolism, detoxification, membrane transport, genome maintenance, and defense-related responses.

To further prioritize candidate genes, the 247 interval genes were screened according to SNP location, allelic effects on DSI, and functional annotations related to plant defense or stress responses. Six genes were retained as putative candidates for FGRR resistance: *Glyma.09g168300*, *Glyma.12g220200*, *Glyma.17g202000*, *Glyma.17g202500*, *Glyma.18g033900*, and *Glyma.18g266700*. These genes contained exonic SNPs associated with DSI variation and were therefore selected for further allelic-effect analysis.

### 2.4. Allelic Effects of Exonic SNPs in Candidate Genes

**Figure 4:**
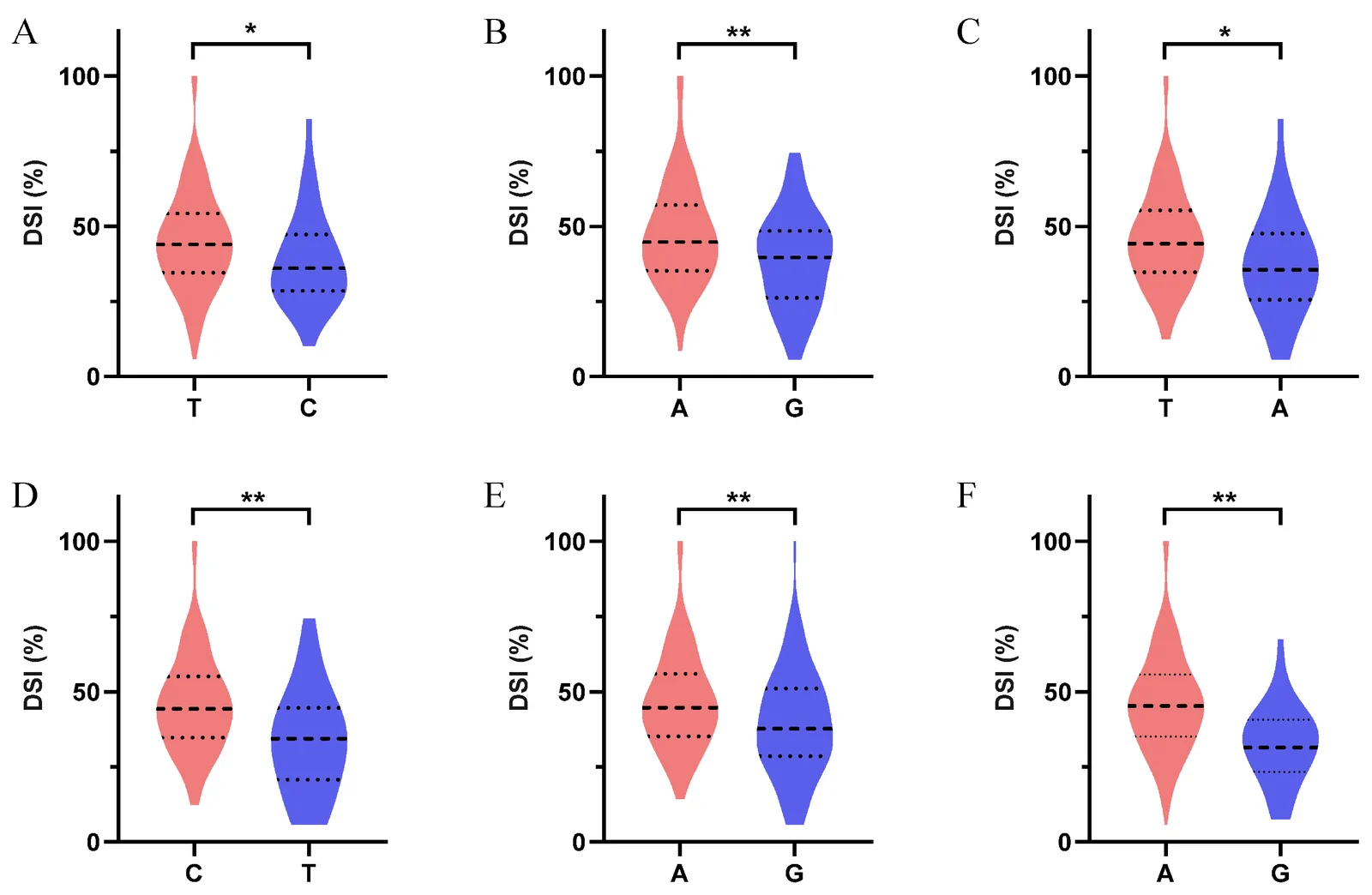
Associations of candidate exonic SNPs with DSI after *Fusarium graminearum* inoculation. (**A**) S09_39290818 (T/C); (**B**) S12_37955440 (A/G); (**C**) S17_32166476 (T/A); (**D**) S17_32244510 (C/T); (**E**) S18_2618132 (A/G); and (**F**) S18_55105706 (A/G). Violin plots show the distribution of DSI values in accessions carrying different alleles. Dashed lines indicate the median and interquartile range. Differences between allelic classes were tested using Student’s *t*-test (* *p* < 0.05, ** *p* < 0.01).

To further examine the allelic effects of exonic SNPs within the candidate genes, DSI values were compared among accessions carrying different alleles at each locus (Table 1; Figure 4). The two allele classes showed significantly different mean DSI values at each of the six SNPs. Among them, four SNPs showed highly significant differences (*p* < 0.01), whereas the remaining two showed significant differences (*p <* 0.05). For each SNP, one allele was associated with a lower mean DSI and was therefore defined as the favorable allele in this panel. The favorable alleles were C at S09_39290818, G at S12_37955440, A at S17_32166476, T at S17_32244510, G at S18_2618132, and G at S18_55105706.

At these loci, accessions carrying the favorable alleles had 6.66–14.07 lower mean DSI values than those carrying the corresponding alternative alleles. The largest difference was observed at S18_55105706 in *Glyma.18g266700*, where accessions carrying the G allele showed a mean DSI 14.07 lower than those carrying the A allele. A relatively large difference was also detected at S17_32244510 in *Glyma.17g202500*. At this locus, accessions carrying the T allele had a mean DSI 12.78 lower than those carrying the C allele. These results further support the association between these exonic SNPs and DSI differences among soybean accessions.

### 2.5. Transcriptional Response of Candidate Genes to FGRR Inoculation

**Figure 5:**
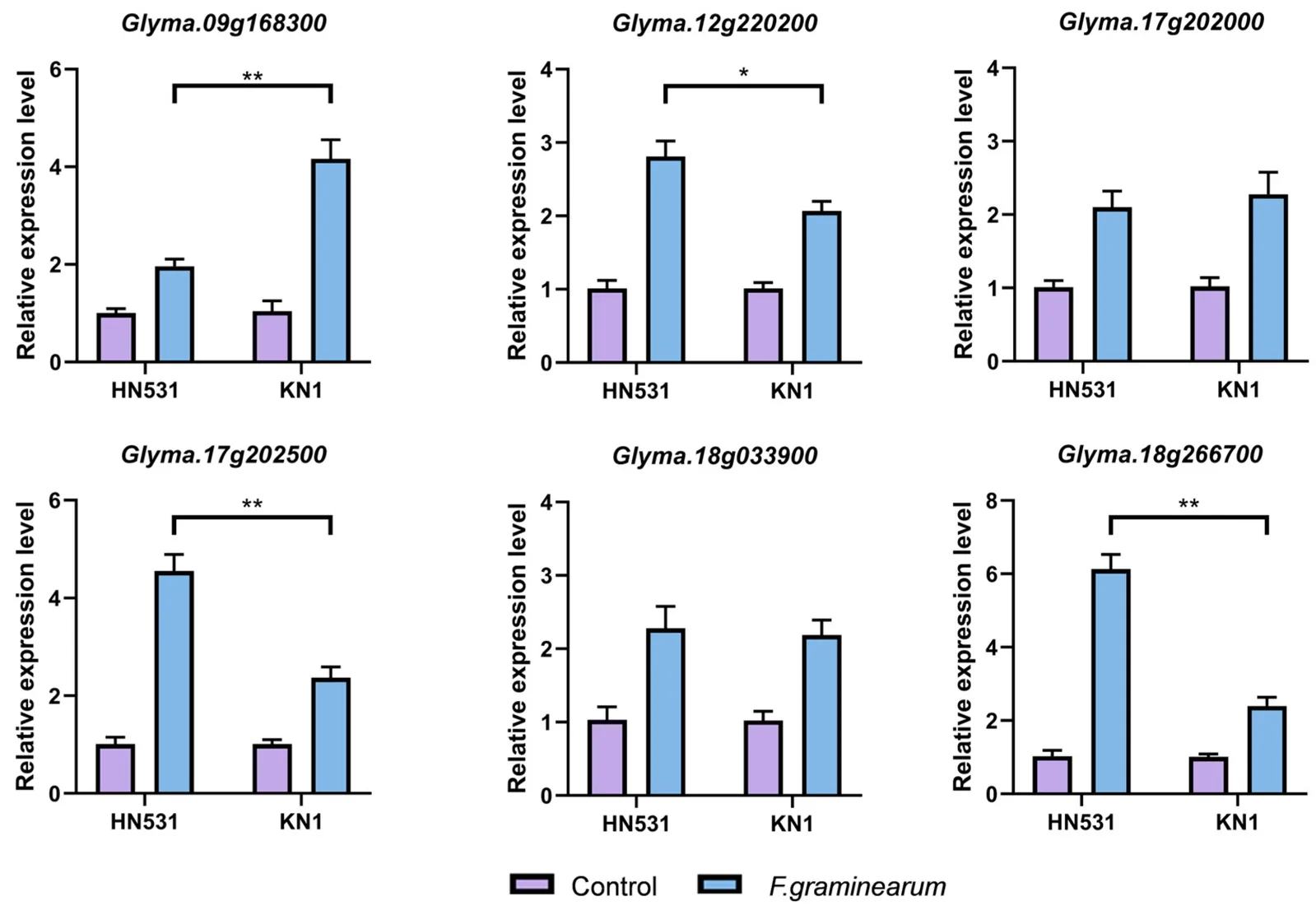
Relative transcript abundance of six candidate genes was compared between the resistant accession HN531 and the susceptible accession KN1 following *F. graminearum* inoculation. Values represent the mean ± SE from three biological replicates. For each gene, significant differences between HN531 and KN1 in inoculation-induced relative expression changes are indicated by asterisks (* *p* < 0.05, ** *p* < 0.01).

To determine whether these candidate genes responded to *F. graminearum* infection, qRT-PCR was performed at 72 h post-inoculation in HN531 and KN1, representing resistant and susceptible genotypes, respectively. Mock-inoculated seedlings served as controls. All six candidate genes were induced after inoculation, but their expression patterns differed between HN531 and KN1 (Figure 5). Compared with the mock treatment, *Glyma.12g220200*, *Glyma.17g202500*, and *Glyma.18g266700* showed stronger induction in HN531 than in KN1, whereas *Glyma.09g168300* showed higher induction in KN1. Among these genes, *Glyma.17g202500* and *Glyma.18g266700* showed the clearest expression differences between the resistant and susceptible genotypes after infection. This result was consistent with the GWAS signals and the DSI differences observed between allelic classes. *Glyma.17g202000* and *Glyma.18g033900* were also induced by *F. graminearum*, but their expression differences between HN531 and KN1 were relatively small. Overall, the qRT-PCR results showed that several GWAS-derived candidate genes responded to *F. graminearum* infection, with stronger induction of selected genes in the resistant genotype. These results provided a basis for selecting SNP targets for subsequent KASP assay development.

### 2.6. Development and Evaluation of KASP Assays for FGRR Resistance

**Figure 6:**
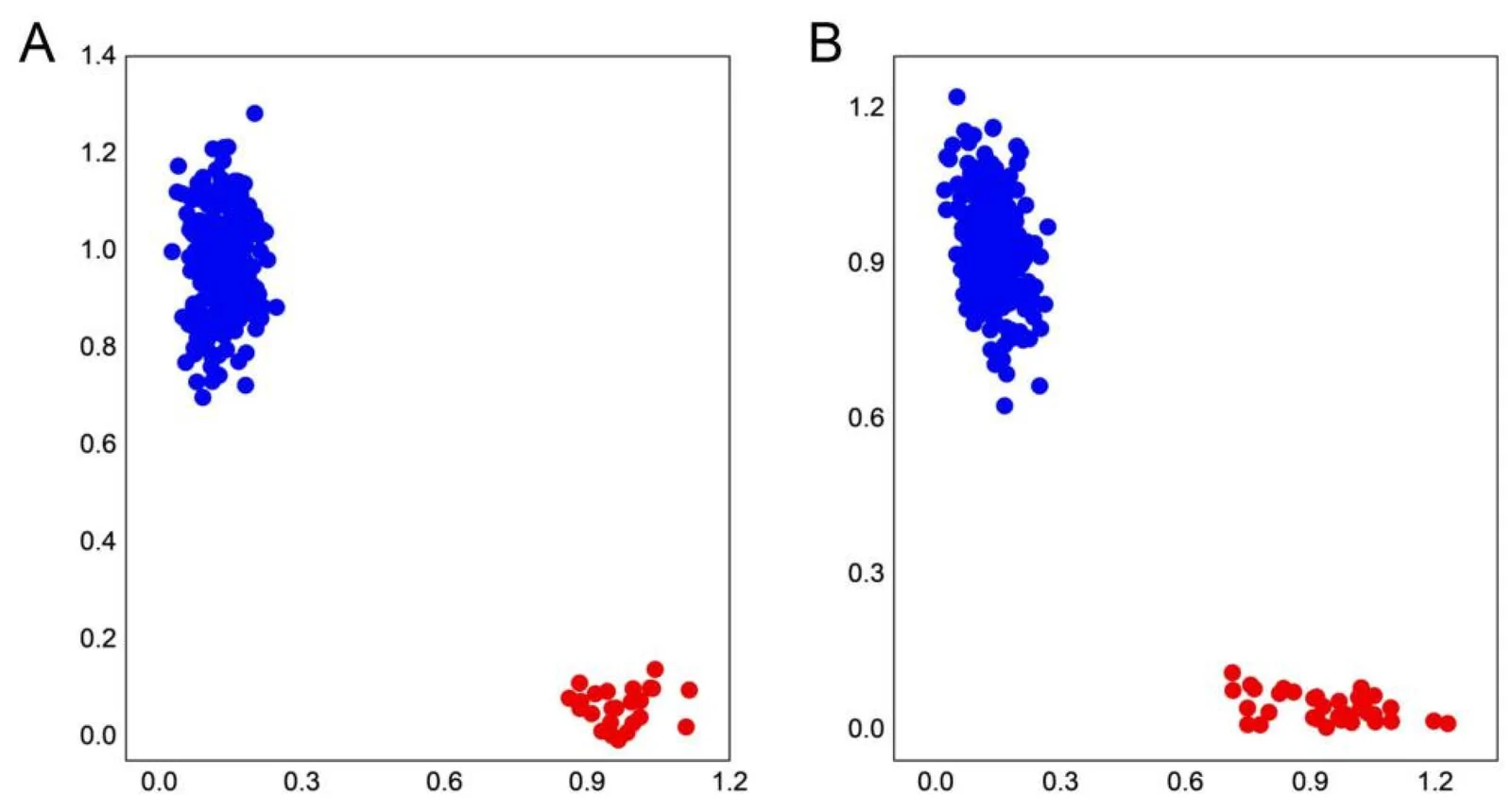
Validation of two SNP markers for FGRR resistance using KASP assays. (**A**) Allelic discrimination plot of KASP-S17_32244510 for the S17_32244510 (C/T) SNP in *Glyma.17g202500*. (**B**) Allelic discrimination plot of KASP-S18_55105706 for the S18_55105706 (A/G) SNP in *Glyma.18g266700*. The screening efficiencies of KASP-S17_32244510 and KASP-S18_55105706 were 73.08% and 74.29%, respectively, calculated as the proportion of accessions with DSI ≤ 40 among those carrying the corresponding favorable genotypes. In (**A**), the blue and red clusters represent the CC and TT homozygous genotypes, respectively. In (**B**), the blue and red clusters represent the AA and GG homozygous genotypes, respectively.

Among the six candidate loci, S17_32244510 and S18_55105706 were selected for KASP assay development. At both loci, the favorable alleles were associated with significantly lower DSI values, and the corresponding genes, *Glyma.17g202500* and *Glyma.18g266700*, showed stronger infection-induced expression in the resistant accession than in the susceptible accession. KASP-S17_32244510 was designed for the C/T variant in *Glyma.17g202500*, and KASP-S18_55105706 was designed for the A/G variant in *Glyma.18g266700*. Both markers produced clear allelic discrimination patterns. The homozygous accessions were separated into two distinct groups, indicating that the two target SNPs were successfully converted into KASP assays for genotype calling (Figure 6A,B).

The screening performance of each marker was assessed using the DSI-based resistance classification as the phenotypic reference. For KASP-S17_32244510, 26 accessions carried the favorable TT genotype, of which 19 had DSI values ≤ 40 and were therefore classified as resistant, resulting in a screening efficiency of 73.08%. For KASP-S18_55105706, 35 accessions carried the favorable GG genotype, of which 26 had DSI values ≤ 40 and were therefore classified as resistant, giving a screening efficiency of 74.29%. These values exceeded the overall proportion of resistant accessions in the association panel, suggesting that the favorable genotypes were enriched in resistant germplasm. Together, these results show that the two KASP markers effectively enriched resistant accessions and can serve as practical tools for rapid screening of FGRR resistance in soybean germplasm.

To further examine the joint effects of the two KASP markers, 308 accessions with unambiguous homozygous genotype calls at both loci were grouped into four genotype combinations (Supplementary Table S6). In the two-locus linear regression analysis, both marker terms showed significant associations with DSI, whereas the interaction term was not statistically significant (*p* = 0.095). Mean DSI values for the CC/AA, TT/AA, CC/GG, and TT/GG combinations were 47.21, 37.41, 35.21, and 9.98, respectively, with the TT/GG class, represented by four accessions, showing the lowest mean DSI.

## 3. Discussion

*Fusarium graminearum* root rot has become an increasing threat to soybean production, especially in temperate regions where maize-soybean rotation is widely practiced [31]. Breeding for FGRR resistance is still limited by the shortage of resistant germplasm and the lack of effective molecular markers [30]. This limitation is particularly important because resistance to soybean diseases, including root rot diseases, often involves multiple QTLs, genes, and resistance-associated alleles [32]. Therefore, identifying resistance-associated loci and developing linked markers are necessary steps for improving germplasm evaluation and marker-assisted selection [33]. In this study, the 336 soybean accessions showed clear phenotypic variation in disease response, indicating that the panel contained useful genetic diversity for resistance analysis. By combining GWAS, candidate-gene analysis, expression evidence, and KASP marker development, we detected genomic regions associated with FGRR resistance and developed two marker assays for potential use in germplasm screening. These results provide additional genetic information and practical marker resources for soybean FGRR resistance improvement and also offer candidate targets for future functional studies.

Careful phenotypic assessment is essential for screening resistant germplasm. It also provides the foundation for elucidating the genetic basis of FGRR resistance [34]. DSI values displayed a continuous and nearly unimodal distribution. This trend indicates that FGRR resistance is likely determined by the combined effects of several loci rather than one major gene [10]. Comparable disease-response patterns have also been observed in other soybean panels evaluated for *F. graminearum* [17]. No immune accession was detected, and only 5.06% of the accessions were classified as highly resistant. This finding is consistent with earlier reports showing that complete immunity to *F. graminearum* and related *Fusarium* species is uncommon in cultivated soybean germplasm [30]. The low proportion of highly resistant materials highlights the difficulty of improving FGRR resistance through conventional selection alone. This difficulty is further compounded by the persistence of *Fusarium* pathogens in soil and crop residues across successive growing [13,35]. The highly resistant accessions identified from this panel may serve as valuable donor germplasm. They could be used to breed soybean cultivars with enhanced FGRR resistance. They may serve as resistant parents in breeding programs and as useful materials for future studies on the genetic and physiological mechanisms underlying FGRR resistance.

The continuous distribution of DSI values further supported the use of GWAS to dissect the genetic basis of FGRR resistance. In this study, MLM and FarmCPU detected partially overlapping association signals, and the SNPs identified by both models were used to define candidate regions. Because the two models differ in their statistical assumptions, their combined use can reduce model-specific bias and improve confidence in the detected loci [36,37]. Previous genetic mapping studies have identified several resistance-associated genomic regions for soybean *Fusarium* root rot. For F. graminearum root rot resistance, linkage mapping studies have reported QTLs on chromosomes 6, 8, 13, 15, 16, and 19 using different recombinant inbred line populations [17,18,19]. Among these loci, qRfg_Gm06 on chromosome 6 represents one of the most extensively characterized resistance regions, which was further fine-mapped to an approximately 400 kb interval and used for KASP marker development [7]. A previous SLAF-seq-based GWAS involving 314 soybean accessions also identified resistance-associated SNPs across eight chromosomes, including one locus overlapping with qRfg_Gm06 and several additional candidate regions [10]. In the present study, the main association signals were located on chromosomes 12, 17, and 18, with the most prominent signals observed on chromosome 17. These loci provide additional candidate regions for understanding the genetic basis of FGRR resistance beyond the previously characterized major QTL regions and may offer useful genetic resources for resistance breeding. By contrast, no significant signal was detected near the previously reported major QTL qRfg_Gm06 on chromosome 6. This does not necessarily indicate the absence of this locus in the present population but may reflect differences in allele frequency and genetic background among the tested materials. Differences in resistance-associated loci among studies may reflect variation in mapping populations and soybean genetic backgrounds, allele frequencies, pathogen species or isolates, and disease evaluation systems [10,20,38]. Previous QTL studies were often conducted using biparental populations derived from specific resistant donors, which are effective for identifying resistance loci contributed by particular genetic backgrounds. In contrast, GWAS based on diverse natural populations can capture broader allelic variation across different soybean genetic backgrounds. Therefore, the candidate regions identified in the present study may complement previously reported resistance loci and provide additional information on the genetic basis of FGRR resistance.

Differences in pathogen species and evaluation methods may also contribute to the identification of distinct resistance-associated loci. Sang et al. investigated resistance to *F. oxysporum* root rot in soybean [16], whereas Okello et al. evaluated resistance sources and marker-trait associations for *F. graminearum* root rot [30]. Okello et al. reported six marker–trait associations based on 42,079 SNP markers, with the associated SNPs located in intergenic or untranslated regions [30]. The present study provides complementary information by further evaluating exonic SNPs and their corresponding candidate genes through allelic-effect and expression analyses and by developing KASP assays targeting two selected SNPs for germplasm screening. Wang et al. investigated F. oxysporum root rot resistance using an MLM-based GWAS [39]. Although some soybean germplasm and genotypic resources are shared between the two studies, the present study independently evaluated resistance to F. graminearum and used the resulting FGRR phenotype for association analysis with both MLM and FarmCPU models. These studies suggest that different *Fusarium* pathosystems may lead to the identification of different genetic components associated with host resistance. Similar complexity has been observed in other plant species. Taylor et al. identified resistance to *Fusarium* basal rot and improved seedling vigor in onion, further illustrating the diversity of *Fusarium* resistance across different host–pathogen systems [40]. Therefore, pathogen-specific evaluation may facilitate the identification of additional genomic regions associated with FGRR resistance. In this context, the resistance-associated regions identified in the present study provide additional candidate loci associated with FGRR resistance and complement the genetic information reported in previous studies.

In the present study, the chromosome 17 and 18 regions identified by GWAS represent additional candidate loci associated with FGRR resistance. The relevance of these regions was further supported by candidate-gene analyses, including allelic-effect and expression analyses. These findings complement the previously characterized qRfg_Gm06 region on chromosome 6 and further support the involvement of multiple genomic components in soybean FGRR resistance. Together with the candidate genes *Glyma.17g202500* and *Glyma.18g266700*, these results provide additional genetic information for understanding FGRR resistance and potential resources for future resistance breeding.

Candidate-gene analysis of the GWAS regions provided further insight into the biological basis of FGRR resistance. These candidate intervals contained 247 nonredundant genes. Enrichment analysis linked the candidate genes to glutathione metabolism, isoflavonoid biosynthesis, membrane-associated transport, carbohydrate-related pathways, proteasome function, homologous recombination, and plant–pathogen interaction. These processes are widely recognized as important components of plant defense responses [41,42]. Among the genes carrying exonic SNPs associated with DSI variation, *Glyma.17g202500* and *Glyma.18g266700* were particularly notable. Their corresponding SNPs, S17_32244510 and S18_55105706, showed clear allelic differences in DSI. Accessions carrying their favorable alleles had lower DSI values, and both genes showed stronger induction in the resistant accession at 72 h post-inoculation. Transcriptome profiling has also shown broad transcriptional changes in soybean roots after challenge with pathogenic *Fusarium* isolates [43]. Together with the allele–DSI associations, the induction patterns observed here suggest that *Glyma.17g202500* and *Glyma.18g266700* may be involved in the soybean response to *F. graminearum*. In contrast, *Glyma.09g168300* was more highly expressed in KN1, suggesting a possible role in susceptibility to *F. graminearum*. The remaining three candidate genes showed less consistent or weaker expression differences between the two genotypes, suggesting that their effects may be smaller or more dependent on genetic background or infection stage. Overall, these results indicate that FGRR resistance may involve multiple defense-related processes and genes.

The development of KASP markers further extended the application value of the GWAS results. Previous studies in soybean have also converted resistance- or stress-associated SNPs into KASP assays for genotype discrimination, germplasm screening, and marker-assisted breeding [7,44]. In this study, two exonic SNPs located in *Glyma.17g202500* and *Glyma.18g266700* were converted into KASP markers. Well-separated genotype clusters were formed at both SNP loci, confirming that the corresponding KASP markers resolved the allelic classes [45]. KASP-S17_32244510 and KASP-S18_55105706 showed screening efficiencies of 73.08% and 74.29%, respectively, exceeding the overall proportion of resistant accessions in the association panel. This result suggests that the two markers may be useful for rapid germplasm screening and preliminary selection of favorable alleles [18]. The two-locus analysis showed significant main effects of both markers on DSI, whereas the marker-by-marker interaction was not statistically significant (*p* = 0.095), providing no statistically significant evidence of epistatic interaction. The TT/GG combination showed the lowest mean DSI among the four genotype classes, suggesting a potential advantage of combining the two favorable alleles, although this observation should be interpreted cautiously because the TT/GG class contained only four accessions. Because KASP assays are cost-effective, scalable, and easy to apply in large populations, these markers provide practical tools for marker-assisted screening in soybean breeding programs.

In summary, this study revealed broad phenotypic variation and identified highly resistant accessions in a diverse soybean panel. Using two complementary GWAS models, we detected resistance-associated genomic regions on chromosomes 9, 12, 17, and 18, providing additional loci for understanding the quantitative inheritance of soybean response to *F. graminearum*. Candidate-gene analysis further highlighted defense-related pathways and several genes were supported by allelic-effect and expression evidence, particularly *Glyma.17g202500* and *Glyma.18g266700.* In addition, two associated SNPs were converted into KASP markers, which showed screening efficiencies of 73.08% and 74.29% and may facilitate rapid germplasm screening. Overall, these findings expand the genetic information available for FGRR and provide useful resources for resistance breeding, marker-assisted screening, and future functional studies.

## 4. Materials and Methods

### 4.1. Plant Materials and Fusarium graminearum Isolate

The experimental panel included 336 soybean germplasm accessions. The materials were supplied by the Soybean Research Institute of Northeast Agricultural University (NEAU), Harbin, China. The collection included mainly Chinese landraces and improved cultivars, along with a few accessions from other countries. Plants were grown under controlled greenhouse conditions at NEAU from April to September 2025. Disease response to *F. graminearum* root rot was evaluated under these conditions. The L09 isolate of *F. graminearum* was obtained from the same institute and used for inoculation. The fungal culture was maintained on PDA plates at 4 °C without light exposure until it was used for inoculum production.

### 4.2. Preparation of Fusarium graminearum Inoculum

Wheat-kernel inoculum preparation began with incubation of *F. graminearum* isolate L09 on PDA plates at 25 °C in the dark for 7 days, followed by the procedure described by Zhang et al. [46]. For each batch, 1 kg of wheat kernels was immersed in distilled water for 24 h. The hydrated kernels were then autoclaved at 121 °C and 0.105 MPa for 60 min. Once cooled to room temperature, each bag of kernels was inoculated with twenty 6 mm agar plugs taken from 7-day-old PDA cultures of *F. graminearum*. The inoculated kernels were thoroughly mixed and incubated at 23 °C for 10 days to facilitate fungal colonization. After fungal colonization, the kernels were air-dried and fragmented. The resulting inoculum was mixed with sterile vermiculite at a 1:50 ratio (*v*/*v*). The inoculated substrate was transferred to 300 mL seedling pots. Soybean seeds were then sown directly into the prepared medium.

### 4.3. Disease Phenotyping and Resistance Classification

Disease severity was assessed 10 days after sowing into the infested substrate. Each seedling was assigned a root rot severity score on a modified 0–7 scale adapted from a published protocol [47]. The rating was based on root discoloration, root decay, lateral root development, and overall seedling growth. The detailed criteria for each disease rating are presented in Table 2. For each accession, 20 seeds were sown per replicate. Disease severity was assessed individually, with at least 15 seedlings evaluated in each replicate. Three independent biological replicates were performed. The disease severity index (DSI) was calculated as follows:

$$\mathrm{DSI}=\frac{\Sigma \;(\mathrm{Rating}\;\mathrm{value}\;\times \;\mathrm{Number}\;\mathrm{of}\;\mathrm{plants}\;\mathrm{with}\;\mathrm{this}\;\mathrm{rating})}{\mathrm{Total}\;\mathrm{number}\;\mathrm{of}\;\mathrm{plants}\;\mathrm{surveyed}\;\times \;7}\times 100\%$$

Based on DSI values, the accessions were assigned to four resistance categories: highly resistant (HR, 0 < DSI ≤ 20), resistant (R, 20 < DSI ≤ 40), susceptible (S, 40 < DSI ≤ 80), and highly susceptible (HS, DSI > 80).

### 4.4. SNP Genotyping and Genome-Wide Association Analysis

The SNP markers used for GWAS were derived from previous laboratory resequencing work on a soybean natural population [39]. For the 336 soybean accessions evaluated in this study, SNPs were re-filtered using TASSEL version 5 before association analysis. Markers with MAF < 0.05 or more than 10% missing genotypes were removed. After filtering, 1,329,617 high-quality SNPs were retained for subsequent analysis.

Principal component analysis (PCA) was conducted in GAPIT version 3 to account for population structure [48]. GWAS was then carried out with GAPIT 3.0 in R version 4.5.2. Both FarmCPU and MLM approaches were applied to identify SNPs associated with resistance to *F. graminearum* root rot. Signals with −log_10_(*P*) ≥ 4.0 were considered suggestive and retained for candidate gene analysis [49]. Association signals detected by the two models were compared, and SNPs detected by both models were retained for subsequent interval definition and candidate-gene analysis.

### 4.5. Candidate Gene Identification, Annotation, and Enrichment Analysis

Candidate genes were searched within 100 kb upstream and downstream of each associated SNP. The resulting 200 kb window was defined with reference to a previous LD-decay estimate based on the same soybean resequencing SNP resource, where the average LD distance was approximately 207 kbp [39]. Gene models and functional annotations were obtained from Phytozome using the Wm82.a2.v1 assembly [50]. Genes shared among overlapping candidate intervals were collapsed into a single entry. The resulting nonredundant gene set was used for downstream analyses. Enrichment analysis was performed using Gene Ontology (GO) categories and Kyoto Encyclopedia of Genes and Genomes (KEGG) pathways. Enrichment results with an FDR < 0.05 were regarded as statistically significant. Enrichment significance was visualized in bubble plots using −log_10_(*P*-value).

### 4.6. qRT-PCR Analysis of Candidate Gene Expression

To evaluate candidate gene expression in response to *F. graminearum* infection, two soybean accessions with contrasting disease responses were selected for qRT-PCR analysis. HN531 and KN1 were selected as the resistant and susceptible genotypes, respectively, according to their disease responses after inoculation. Seeds of both accessions were germinated in sterilized vermiculite at 25 °C with a 12 h light/12 h dark cycle. The *F. graminearum* isolate L09 was cultured on PDA plates. Conidia were gently washed from the colony surface using sterile distilled water. The suspension was filtered through sterile gauze to eliminate hyphal debris. Spore concentration was adjusted to 1 × 10^6^ spores mL^−1^ after hemocytometer counting. Four-day-old seedlings were inoculated with the spore suspension, and mock-treated seedlings received sterile distilled water. Roots were harvested at 72 hpi. Samples were snap-frozen in liquid nitrogen and kept at −80 °C before RNA extraction.

Taproot tissues were ground to a fine powder in liquid nitrogen before RNA isolation. Soybean taproot tissue was processed for RNA extraction with the FastPure Universal Plant Total RNA Isolation Kit (Vazyme, Nanjing, China), according to the manufacturer’s protocol. RNA quality was evaluated by spectrophotometric measurement and 0.8% agarose gel electrophoresis before cDNA synthesis. For qRT-PCR analysis, 1 μg of soybean taproot RNA was converted to first-strand cDNA using BeyoRT™ III cDNA Synthesis Master Mix (Beyotime, Shanghai, China). qRT-PCR was conducted using an ABI 7500 system with SYBR-based detection chemistry. The amplification protocol included 95 °C for 30 s, followed by 40 cycles of 95 °C for 5 s and 60 °C for 30 s. Product specificity was checked by melting-curve analysis from 60 °C to 95 °C. Relative gene expression was normalized to soybean Actin4 and calculated using the 2^−ΔΔCt^ method. Three biological replicates were analyzed for each treatment, with three technical repeats per sample. Details of the primers are given in Supplementary Table S7.

### 4.7. Development and Screening Efficiency Analysis of Trait-Associated KASP Assays

The genomic sequences of *Glyma.17g202500* and *Glyma.18g266700* were retrieved from the Phytozome database. Two SNPs, S17_32244510 (C/T) in *Glyma.17g202500* and S18_55105706 (A/G) in *Glyma.18g266700*, were selected for KASP assay development because they were detected by GWAS and showed significant allelic effects on DSI. For each SNP, representative accessions carrying the two homozygous genotypes were used to confirm allele-specific amplification and cluster separation. KASP primers were designed using the flanking sequences of each target SNP. Their sequences are provided in Supplementary Table S7, and sequence alignments around S17_32244510 and S18_55105706 are presented in Supplementary Figure S2. For each target SNP, the KASP primer set consisted of two allele-specific forward primers and one universal reverse primer. The 3′ terminal base of each allele-specific primer corresponded to one of the two alleles at the target SNP, and standard FAM- or HEX-compatible tails were added for fluorescence-based allelic discrimination. For S17_32244510, the C allele was labeled with FAM and the T allele with HEX. For S18_55105706, the A allele was labeled with FAM and the G allele with HEX. Primer specificity was checked using the NCBI Primer-BLAST web tool (accessed on 25 March 2026), and primers with predicted nonspecific amplification were excluded.

KASP genotyping was carried out on an ABI 7500 Real-Time PCR System using KASP V4.0 2× Master Mix. Reactions were first activated at 94 °C for 15 min. Ten touchdown cycles followed, each consisting of 94 °C for 20 s and 65–57 °C for 60 s, with the annealing temperature reduced by 0.8 °C per cycle. Amplification was then continued for 35 cycles at 94 °C for 20 s and 57 °C for 60 s. Endpoint fluorescence from the FAM and HEX channels was analyzed with ABI 7500 software v2.0.6. Accessions with DSI values ≤ 40 were considered resistant, and marker screening efficiency was calculated as the percentage of resistant accessions among those carrying the favorable genotype.

### 4.8. Two-Locus Analysis of KASP Markers

To evaluate the joint effects of the two developed KASP markers, a two-locus analysis was performed using KASP genotype calls at S17_32244510 and S18_55105706. Of the 336 accessions in the association panel, 308 had unambiguous homozygous genotype calls at both loci and were included in this analysis. Mean DSI values were summarized for the four two-locus genotype combinations (CC/AA, TT/AA, CC/GG, and TT/GG). The main effects of the two marker genotypes and their interaction were further evaluated using a linear regression model containing terms for S17_32244510, S18_55105706, and their interaction, following the SNP–SNP interaction framework described by Moellers et al. [51]. Statistical significance of the main and interaction terms was assessed at *p* < 0.05.

## 5. Conclusions

This study evaluated seedling resistance to *F. graminearum* root rot in 336 soybean accessions, and DSI values showed a continuous distribution with broad phenotypic variation across the panel. Using MLM and FarmCPU, GWAS detected resistance-associated signals across multiple chromosomes. The 105 SNPs shared by the two models were used to define candidate genomic intervals, from which 247 annotated genes were retrieved. Considering the functional enrichment results, allelic effects, gene annotation, and expression responses, six genes harboring exonic SNPs were prioritized as candidates. The favorable alleles of exonic SNPs in *Glyma.17g202500* and *Glyma.18g266700* were linked to lower DSI values, and the stronger induction of both genes in the resistant accession suggests their possible involvement in the soybean response to *F. graminearum.* Two SNPs in these genes were further converted into KASP markers. KASP-S17_32244510 and KASP-S18_55105706 showed screening efficiencies of 73.08% and 74.29%, respectively. These results provide useful candidate gene resources and molecular markers for marker-assisted screening of soybean resistance to *F. graminearum* root rot.

## Figures and Tables

**Table 1:** Allele-associated DSI variation for candidate SNPs after *Fusarium graminearum* inoculation.

SNP	Candidate Gene	Region	Allele	Mean DSI	ΔDSI
S09_39290818	*Glyma.0* *9* *g* *168300*	Exon	T(REF)	45.30	−6.66
			C(ALT)	38.64	
S12_37955440	*Glyma.12g220200*	Exon	A(REF)	47.58	−8.77
			G(ALT)	38.82	
S17_32166476	*Glyma.17g202000*	Exon	T(REF)	45.96	−8.82
			A(ALT)	37.15	
S17_32244510	*Glyma.17g202500*	Exon	C(REF)	45.97	−12.78
			T(ALT)	33.19	
S18_2618132	*Glyma.18g033900*	Exon	A(REF)	46.78	−7.78
			G(ALT)	39.00	
S18_55105706	*Glyma.18g266700*	Exon	A(REF)	46.40	−14.07
			G(ALT)	32.33	

Mean DSI was estimated for each allele class. REF and ALT denote the reference and alternative alleles, respectively. ΔDSI was calculated as the mean DSI of the ALT class minus that of the REF class.

**Table 2:** Visual rating scale for *Fusarium graminearum* root rot symptoms used for DSI calculation.

Disease Score	Symptom Criteria
0	Healthy seedlings with no visible symptoms.
1	Faint discoloration of the taproot; lateral root development remained normal.
3	Taproot or lateral roots showing dark-brown lesions, with slight inhibition of seedling growth.
5	Extensive browning of the taproot and distinct lesions on lateral roots; shoot growth was markedly restricted.
7	Severe decay or complete breakage of the taproot, accompanied by extensive blackening and decay of the lateral roots. Seedlings showed severe wilting or died.

## Data Availability

Data are available in the manuscript and in the Supplementary Materials.

## Supplementary Materials

The following supporting information can be downloaded at: https://www.mdpi.com/article/10.3390/plants15162479/s1, Supplementary Figure S1: Population structure and genetic relatedness of the soybean association panel; Supplementary Figure S2: Flanking-sequence alignment for the KASP marker loci S17_32244510 and S18_55105706; Supplementary Table S1: FGRR resistance profiles of the 336 soybean accessions; Supplementary Table S2: Genome-wide distribution of SNP markers retained after filtering; Supplementary Table S3: DSI-associated SNPs detected using the MLM; Supplementary Table S4: DSI-associated SNPs detected using the FarmCPU model; Supplementary Table S5: Candidate genes identified near FGRR resistance-associated peak SNPs; Supplementary Table S6: DSI values of soybean accessions carrying different genotype combinations of KASP-S17_32244510 and KASP-S18_55105706; Supplementary Table S7: Primers for qRT-PCR validation and KASP genotyping.
