# Supplementary material for: Association Mapping of Seedling Resistance to Fusarium graminearum Root Rot and Development of KASP Assays in Soybean
Source: Plants (Basel). 2026 Aug 16;15(16):2479. doi: 10.3390/plants15162479 (PMC13516154; doi:10.3390/plants15162479)
Supplement: Supplementary file 1 [file plants-15-02479-s001.zip › Supplementary Figure S1.pdf]

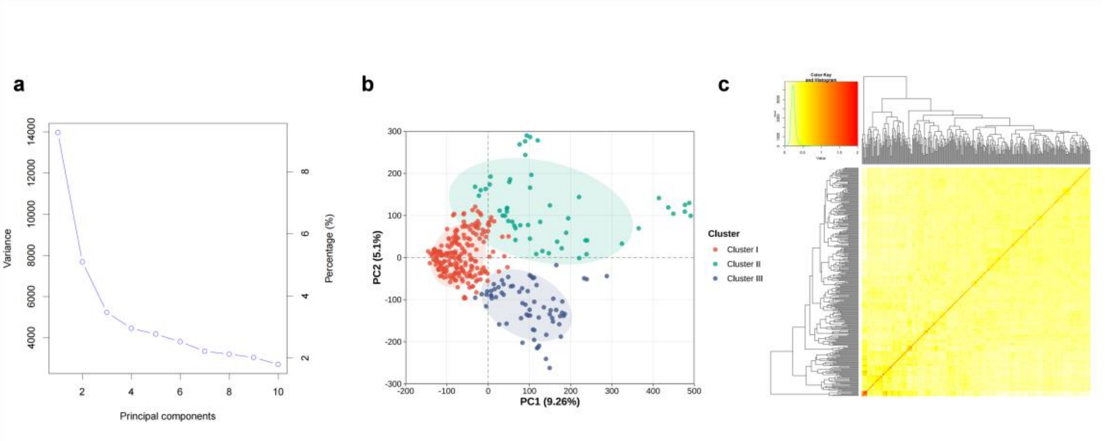

Supplementary Figure S1: Population structure and genetic relatedness of the soybean association panel. **(a)** Scree plot showing the eigenvalues and percentages of variance explained by the first ten principal components. **(b)** Two-dimensional PCA plot based on PC1 and PC2, accounting for 9.26% and 5.1% of the total genetic variation, respectively. Each dot represents one accession, with colors indicating the three genetic clusters. Shaded ellipses illustrate the distribution of accessions within each cluster. **(c)** Heatmap of pairwise kinship coefficients, with hierarchical clustering dendrograms displayed along both axes. The color gradient represents the relative degree of genetic relatedness among accessions.
