# Supplementary material for: Association Mapping of Seedling Resistance to Fusarium graminearum Root Rot and Development of KASP Assays in Soybean
Source: Plants (Basel). 2026 Aug 16;15(16):2479. doi: 10.3390/plants15162479 (PMC13516154; doi:10.3390/plants15162479)
Supplement: Supplementary file 1 [file plants-15-02479-s001.zip › Supplementary Figure S2.pdf]

(A)

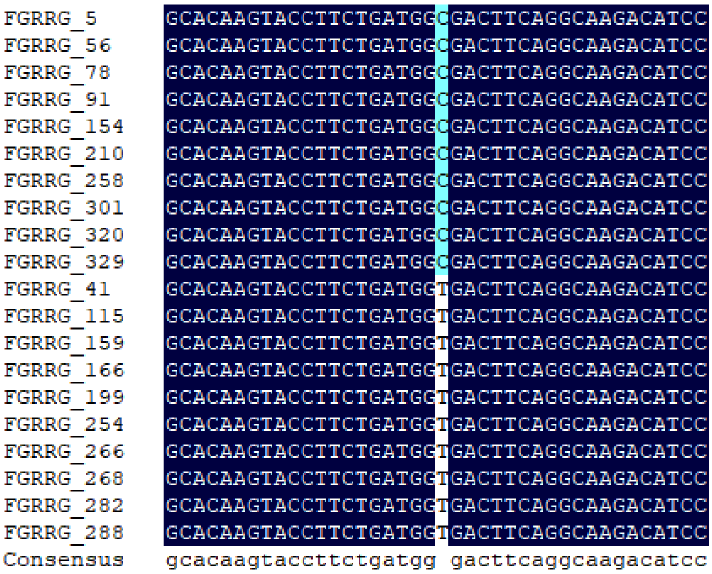

(B)

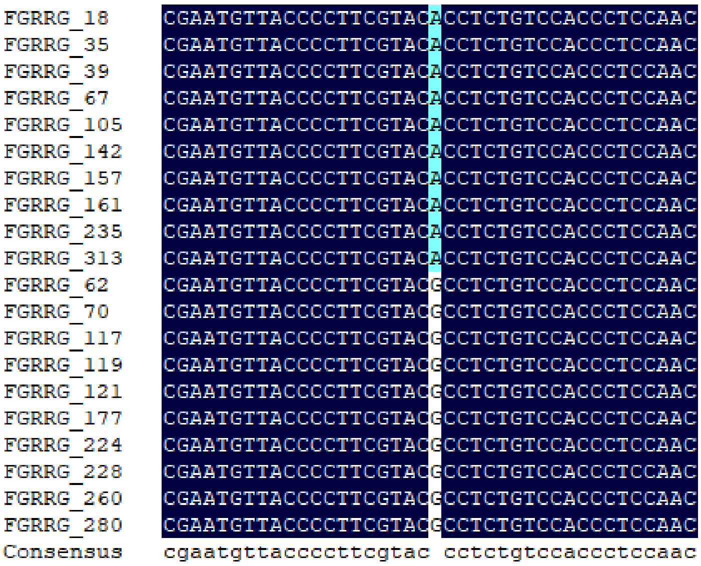

Supplementary Figure S2: Flanking-sequence alignment for the KASP marker loci. S17_32244510 and S18_55105706. (A) S17_32244510 sequence. (B) S18_55105706 sequence.
